# Supplementary material for: Mismatch repair deficiency is not sufficient to elicit tumor immunogenicity
Source: Nat Genet. 2023 Sep 14;55(10):1686–95. doi: 10.1038/s41588-023-01499-4 (PMC10562252; doi:10.1038/s41588-023-01499-4)
Supplement: Supplementary file 1 — Reporting Summary [file 41588_2023_1499_MOESM1_ESM.pdf]

nature portfolio

Corresponding author(s): Tyler Jacks, Peter Westcott, Isidro Cortes-Ciriano

Last updated by author(s): Jul 2, 2023

Reporting Summary

Nature Portfolio wishes to improve the reproducibility of the work that we publish. This form provides structure for consistency and transparency in reporting. For further information on Nature Portfolio policies, see our Editorial Policies and the Editorial Policy Checklist.

Statistics

For all statistical analyses, confirm that the following items are present in the figure legend, table legend, main text, or Methods section.

n/a	Confirmed	
☐	☒	The exact sample size (*n*) for each experimental group/condition, given as a discrete number and unit of measurement
☐	☒	A statement on whether measurements were taken from distinct samples or whether the same sample was measured repeatedly
☐	☒	The statistical test(s) used AND whether they are one- or two-sided<br>*Only common tests should be described solely by name; describe more complex techniques in the Methods section.*
☐	☒	A description of all covariates tested
☐	☒	A description of any assumptions or corrections, such as tests of normality and adjustment for multiple comparisons
☐	☒	A full description of the statistical parameters including central tendency (e.g. means) or other basic estimates (e.g. regression coefficient) AND variation (e.g. standard deviation) or associated estimates of uncertainty (e.g. confidence intervals)
☐	☒	For null hypothesis testing, the test statistic (e.g. *F*, *t*, *r*) with confidence intervals, effect sizes, degrees of freedom and *P* value noted<br>*Give P values as exact values whenever suitable.*
☒	☐	For Bayesian analysis, information on the choice of priors and Markov chain Monte Carlo settings
☒	☐	For hierarchical and complex designs, identification of the appropriate level for tests and full reporting of outcomes
☒	☐	Estimates of effect sizes (e.g. Cohen's *d*, Pearson's *r*), indicating how they were calculated

Our web collection on statistics for biologists contains articles on many of the points above.

Software and code

Policy information about availability of computer code

Data collection	µCT data were captured using a GE eXplore CT 120 system. Endoscope images were captured using a Karl Storz AIDA HD capture system.<br><br>Flow cytometry data were collected on a BD LSRFortessa using BD FACSDiva v8.0 software.
Data analysis	A custom pipeline was constructed for somatic SNV and indel calling using whole-exome sequencing data employing the following published algorithms: BWA-MEM v0.7.17-r1188, Genome Analysis Toolkit (GATK) v4.1.8.0, MuSE v1.0rc, VarDict v1.8.2, Strelka2 v2.9.2, and SomaticCombiner v1.03. Microsatellite contexts of mutations were annotated using SciRoKo v3.4. Copy number aberrations were detected using FreeBayes v1.3 and PureCN v1.16.0, and cancer cell fraction was estimated using the package cDriver in R v4.0.2. Mutational signature analysis was performed using the R (v4.0.2) package MutationalPatterns71 (v3.2.0) and COSMIC Mutational Signatures catalogue version 3. We used the function fit_to_signatures with default parameter values to estimate the contribution of each mutational process to the observed mutational spectrum in sample group. For fitting the signatures, we only used the mutational processes known to be operative in human colon and/or lung cancer, except for tobacco smoking: SBS1, SBS5, SBS6, SBS10a, SBS10b, SBS14, SBS15, SBS17a, SBS17b, SBS18, SBS21, SBS26, SBS28, SBS37, SBS40, and SBS44. For visualization purposes, we collapsed the contribution of mutational signatures associated with defective DNA mismatch repair (SBS6, SBS14, SBS15, SBS21, SBS26 and SBS44; labelled as MMRd in the figures) and POLE deficiency (SBS10a, SBS10b, SBS28, SBS17b; labelled as POLE in the figures). MSI status of samples was calculated using the software MSIsensor-pro (v1.2.0) with matched normal tails as controls. Mouse tumor purity estimates based on Trp53-flox allele recombination and Msh2-flox allele recombination efficiency were calculated using SAMtools v1.10 and a custom script in R v4.0.2.<br><br>A custom pipeline was constructed for neoantigen prediction employing the following published algorithms: Ensembl Variant Effect Predictor (VEP) v99, pVACtools v1.5.7, NetMHC-4.0, NetMHCpan-4.0, SMM v1.0, SMMPMBEC v1.0, and custom scripts in Python v2.7.13 as previously described (Westcott, PMK, et al., Nature Cancer, 2021 in press). Expression was calculated from RNA sequencing data using STAR v2.7.1a and Picard v2.23.4, and allele-specific expression of mutations associated with predicted neoantigens in the RNA sequencing BAMs was calculated using custom scripts in Python v2.7.13.

Clonal deconvolution of targeted amplicon sequencing data was performed using the following published algorithms: BWA-MEM v0.7.17-r1188, bcftools v1.10.2, and custom scripts in R v4.0.2 and Python v2.7.13. or CleanPlex UMI libraries, fgbio (v2.0.1) was used to extract UMIs (ExtractUmisFromBam) and call consensus reads (GroupReadsByUmi, CallMolecularConsensusReads).

Tumor burden and grade of whole lung H&E sections and triple IHC staining (CD8, CD4, FOXP3) of histological sections were quantified using custom convoluted neural network developed with Aiforia's cloud-based image analysis platform. This is a commercial platform with proprietary technology and therefore did not generate any code. An interactive example of algorithm functionality can be provided free-of-charge upon request at https://www.aiforia.com. MSH2 staining of lung tumors was quantified by manual annotation with QuPath v0.1.2. Quantification of CD3 staining in tumors of lung histological sections was performed with Aperio ImageScope v12.1.

Longitudinal change in lung tumor burden as measured by µCT was calculated using a custom MATLAB (MathWorks) script, as previously described (Tammela, T, et al., Nature 2017). Colon tumor area was calculated from longitudinal endoscope and endpoint stereoscope images using ImageJ v2.1.0/1.53c.

Flow cytometry results were analyzed in FlowJo v10.4.2.

Tandem mass spectra were searched with Sequest (Thermo Fisher Scientific, San Jose, CA, USA; version IseNode in Proteome Discoverer 2.5.0.400). Sequest was set up to search a mouse uniprot database (database v.July 3, 2020; 55650 entries containing common contaminants and the proteins GFP, Cas9, Puromycin, and P2A (present in the cell lines) assuming no digestion enzyme (unspecific).

Statistical analyses and figure generation were performed in R (v4.2.1) using built in functions and ggplot2 (v3.4.1), beeswarm (v0.4.0), corrplot (v0.88), eulerr (v6.1.0), gplots (v3.1.3), survival (v3.4.0), survminer (v0.4.9), and RColorBrewer (v1.1.3).

All custom code used in this study are available from the authors upon request.

For manuscripts utilizing custom algorithms or software that are central to the research but not yet described in published literature, software must be made available to editors and reviewers. We strongly encourage code deposition in a community repository (e.g. GitHub). See the Nature Portfolio guidelines for submitting code & software for further information.

Data

Policy information about availability of data

All manuscripts must include a data availability statement. This statement should provide the following information, where applicable:

- Accession codes, unique identifiers, or web links for publicly available datasets
- A description of any restrictions on data availability
- For clinical datasets or third party data, please ensure that the statement adheres to our policy

Raw exome sequencing and RNA-seq data from Bortolomeazzi et al. (48) are available through controlled-access application via the European Genome-Phenome Archive (EGA, hosted by the EMBL-EBI and the CRG) under the accession number EGAD00001006165. Raw sequencing data from Kwon et al. (35) were downloaded from the European Nucleotide Archive (ENA) database under primary accession number PRJEB40416. The sequencing data generated in this study are available at ENA under primary accession number PRJEB56609. Raw mass spectrometry data generated in this study are available at MassIVE under accession number MSV000092096.

Field-specific reporting

Please select the one below that is the best fit for your research. If you are not sure, read the appropriate sections before making your selection.

☒ Life sciences ☐ Behavioural & social sciences ☐ Ecological, evolutionary & environmental sciences

For a reference copy of the document with all sections, see nature.com/documents/nr-reporting-summary-flat.pdf

Life sciences study design

All studies must disclose on these points even when the disclosure is negative.

Sample size	No sample size power calculations were performed. Numbers were chosen based on a combination of past experience with similar studies and practicality (e.g., available animals, funds), with the aim of including at least 10 animals in each group. In the orthotopic transplant studies, this was not possible due to the large number of groups and treatments and the prolonged nature of survival studies. Therefore, we aimed to include at least 5 animals within each group in these studies. Orthotopic transplant survival studies were also performed on a rolling basis as mice became available, given the large number of animals used in these studies. Preclinical treatment studies were performed across two independent cohorts in both the lung and colon models with the aim of validating consistency of results.
Data exclusions	Only mice with discrete lung tumors by µCT at 10 weeks and mice with clear mScarlet positive (red fluorescent) colon tumors by colonoscopy at 20 weeks were recruited into treatment arms. No other data were excluded.
Replication	All in vivo experiments were repeated at least 2-3 times (as described in Sample size and Data exclusions above) to verify reproducibility. In vitro growth kinetics and IFN gamma sensitivity experiments with cell lines were performed twice. No experiments presented in this manuscript failed to replicate. Whole-exome (WES), targeted amplicon, and RNA sequencing were performed once per animal in two separate batches for RNA-seq and targeted amplicon sequencing and four separate batches for WES. No significant batch effects were observed.
Randomization	Randomization was performed in assigning mice to treatment arms in preclinical trials. µCT was performed at 10 weeks and colonoscopy at 20 weeks and mice without discrete tumors excluded. Mice of each sex (approximately equal numbers) were then randomly and evenly (as

possible) assigned across treatment arms using the Sample function in R v4.0.2. Randomization was not appropriate for other experiments presented in this manuscript as mice were age-, litter-, and sex-matched for consistency, and downstream treatment, sampling, and endpoints were identical across all animals. Only male recipient mice were used in orthotopic transplant experiments, as the tumor cell lines transplanted all carry the Y chromosome.

Blinding

Investigators were blinded to genotypes and treatment groups when dosing during preclinical trials, performing µCT and colonoscopy, quantifying tumor burden from colonoscopy images, stereoscope images, and H&E histological lung sections, and quantifying CD3 infiltration by immunohistochemistry. Quantification of µCT data, H&E tumor burden in the KPM cohort and preclinial trial, and infiltration of CD4, CD8, and Regulatory T cells was performed algorithmically and thus was intrinsically blinded. All other analyses where statistical comparisons were made between different groups were performed blinded.

Reporting for specific materials, systems and methods

We require information from authors about some types of materials, experimental systems and methods used in many studies. Here, indicate whether each material, system or method listed is relevant to your study. If you are not sure if a list item applies to your research, read the appropriate section before selecting a response.

Materials & experimental systems

n/a	Involved in the study
☐	☒ Antibodies
☐	☒ Eukaryotic cell lines
☒	☐ Palaeontology and archaeology
☐	☒ Animals and other organisms
☒	☐ Human research participants
☒	☐ Clinical data
☒	☐ Dual use research of concern

Methods

n/a	Involved in the study
☒	☐ ChIP-seq
☐	☒ Flow cytometry
☒	☐ MRI-based neuroimaging

Antibodies

Antibodies used

In vivo dosing:
CD4 (GK1.5, BioXCell Cat#: BE0003-1) (see Methods for in vivo dosing)
CD8 (2.43, BioXCell Cat#: BP0061) (see Methods for in vivo dosing)
PD-1 (29F.1A12, BioXCell Cat#: BE0273) (see Methods for in vivo dosing)
CTLA-4 (9H10, BioXCell Cat#: BE0131) (see Methods for in vivo dosing)

Westerns:
MSH2 (D24B5, Cell Signaling Technology Cat#: 2017) 1:1000
MLH1 (EPR3894, Abcam Cat#: ab92312) 1:1000
GAPDH (6C5, Santa Cruz Cat#: sc-32233) 1:5000
β-ACTIN (13E5, Cell Signaling Technology Cat#: 4970) 1:5000

Immunohistochemistry:
MSH2 (polyclonal, Abcam Cat#: ab70270) 1:1000
CD8a (EPR21769, Abcam Cat#: ab217344) 1:1000
FOXP3 (FJK-16s, eBioscience Cat#: 14-5773-82) 1:125
CD4 (EPR19514, Abcam Cat#: ab183685) 1:400
CD3 (polyclonal, Abcam Cat#: ab5690) 1:1000
HRP anti-Rabbit IgG (Vector Cat#: MP-7401) 4 drops
AP anti-Rabbit IgG (Vector Cat#: MP-5401) 4 drops

Flow cytometry:
H-2Kb APC (AF6-88.5.5.3, Thermo Fisher Cat#: 17-5958-82) 1:200
H-2Db FITC (28-14-8, Thermo Fisher Cat#: 11-5999-82) 1:200
PD-L1 PE-Cy7 (10F.9G2, BioLegend Cat#: 124313) 1:200
CD8a BUV395 (53-6.7, BioLegend Cat#: 563786) 1:400
CD3 BV421 (17A2, BioLegend, Cat#: 100227) 1:400
CD44 BV785 (IM7, BioLegend Cat#: 103057) 1:200
CD4 AF647 (RM4-5, BioLegend Cat#: 100530) 1:400
CD4 BV711 (RM4-5, BioLegend Cat#: 100549) 1:200
GZMB PE-CF594 (GB11, BD Biosciences Cat#: 562462) 1:250
TCF1 AF647 (C63D9, CST Cat#: 37636S) 1:200
CD45 BV785 (30-F11, BioLegend Cat#: 103149) 1:200
CD45 APC-eFluor 780 (30-F11, eBioscience, Cat#: 47-0451-82) 1:50

QAYAFLQHL H-2Kb tetramer PE/APC (custom purified disulfide stabilized H-2Kb) 1:200
QAYAFLQHL H-2Kb dextramer PE/APC (easYmer H-2Kb U-Load Dextramer, Immudex, cat#: U-LX42) 1:10
MAVQKFPSL H-2Kb tetramer PE/APC (custom purified disulfide stabilized H-2Kb) 1:200
SVYVYRVL H-2Kb tetramer PE/APC (custom purified disulfide stabilized H-2Kb) 1:200
SVLENFTML H-2Kb tetramer PE/APC (custom purified disulfide stabilized H-2Kb) 1:200
SVLENFTML H-2Db tetramer PE/APC (Flex-T UVX H-2Db, BioLegend, cat#: custom/pre-catalog) 1:50

	AALQNAVTF H-2Db tetramer PE/APC (Flex-T UVX H-2Db, BioLegend, cat#: custom/pre-catalog) 1:50<br>VQIPNGAFI H-2Db tetramer PE/APC (Flex-T UVX H-2Db, BioLegend, cat#: custom/pre-catalog) 1:50<br>TSILLVDEI H-2Db tetramer PE/APC (Flex-T UVX H-2Db, BioLegend, cat#: custom/pre-catalog) 1:50<br>SIINFEKL tetramer PE/APC (custom purified disulfide stabilized H-2Kb) 1:200
Validation	Abcam antibodies against CD3, CD8, CD4, and FOXP3 and optimal dilutions used for IHC were validated by confirming expected staining patterns of T cells in positive control spleen and lymphnode tissues, with minimal background in non-lymphoid tissue. The Abcam MSH2 antibody for IHC was validated using sgMsh2-targeted lungs and colons (containing knockout tumors) and control tissue (normal lung, colon, and spleen, and sgCtl-targeted tumors), and showed the expected pattern of constitutive expression in normal tissue and control tumors, and specific knockout in most sgMsh2-targeted tumors. Note: this polyclonal antibody did not perform consistently across batches, with some staining non-specifically. Each lot had to be tested separately. Western antibodies against MSH2 and MLH1 were validated using sgRNA-targeted knockout cell lines and positive control cell lines (without specific targeting). anti-CD4 (GK1.5, BioXCell Cat#: BE0003-1) and anti-CD8 (2.43, BioXCell Cat#: BP0061) at the indicated in vivo doses (see Methods) showed nearly complete depletion of CD4 and CD8 T cells in our models by flow cytometry of peripheral blood, spleens, draining LNs, and colons following two doses. On target depletion of PD-1 on T cells following treatment with anti-PD-1 (29F.1A12, BioXCell Cat#: BE0273) was confirmed by flow cytometry of peripheral blood (using a different anti-PD-1 clone, RMP1-30) of tumor-bearing mice every week following treatment initiation in a previously published model (Westcott, PMK, et al., Nature Cancer, 2021). All other antibodies were used following manufacturer recommendations or most commonly described dilutions in the literature. For flow cytometry, specificity of all antibodies was confirmed by comparing against fluorescence minus one (FMO) controls.

Eukaryotic cell lines

Policy information about cell lines

Cell line source(s)	Lung tumor cell lines, single cell clones, and the mouse ES cell line 12A2 used in this study were developed by the authors. HEK-293 cells were ordered from ATCC.
Authentication	Authentication of genetic events (MSH2 knockout, MSH2 re-expression) were confirmed during the establishment of lung tumor cell lines as described in the manuscript. Authentication of knock-in alleles in 12A2 was previously confirmed by Southern blot. HEK-293 cells were not authenticated.
Mycoplasma contamination	Lung tumor cell lines, single cell clones, and HEK-293 cells used to generate lentivirus have been routinely tested and confirmed negative for mycoplasma contamination.
Commonly misidentified lines<br>(See ICLAC register)	None

Animals and other organisms

Policy information about studies involving animals; ARRIVE guidelines recommended for reporting animal research

Laboratory animals	Autochthonous colon cancer experiments were performed in pure C57BL/6 R26-Cas9 mice. Autochthonous lung cancer experiments were performed in mixed C57BL/6; 129/SvJ mice (KP; R26-LSL-Cas9) and pure C57BL/6 mice (KP, KP; Msh2-flox/flox). Mice in autochthonous and orthotopic models were 6-12 weeks and 10-16 weeks of age at initiation, respectively. Approximately equal numbers of male and female mice were used in all studies, with the exception of the orthotopic lung transplantation experiments, where only male albino C57BL/6 hosts chimeric for the male ES cell line 12A2 (KP; R26LSL-Cas9, mixed C57BL/6 and 129/SvJ) were used. Mice were housed in a facility with a 12-hour light/12-hour dark cycle with temperatures within 68-72°F and 30-70% humidity.
Wild animals	This study did not involve wild animals.
Field-collected samples	This study did not involve field-collected samples.
Ethics oversight	All mouse work was approved by the Department for Comparative Medicine (DCM) at MIT and the Institutional Animal Care and Use Committee (IACUC)

Note that full information on the approval of the study protocol must also be provided in the manuscript.

Flow Cytometry

Plots

Confirm that:

☒ The axis labels state the marker and fluorochrome used (e.g. CD4-FITC).

☒ The axis scales are clearly visible. Include numbers along axes only for bottom left plot of group (a 'group' is an analysis of identical markers).

☒ All plots are contour plots with outliers or pseudocolor plots.

☒ A numerical value for number of cells or percentage (with statistics) is provided.

Methodology

Sample preparation	Cell lines were grown to 70-90% confluence, trypsinized for 10-15 minutes at 37 degrees, resuspended in media and

Sample preparation	pelleted. Cells were then washed in PBS, filtered, and live/dead stained in PBS. Cells were then resuspended in FACS buffer (see Methods) and surface staining performed on ice for 45 minutes. Samples were then run on the flow cytometer.
Instrument	BD LSR Fortessa Flow Cytometer with 355 nm, 405 nm, 488 nm, and 640 nm excitation lasers.
Software	BD FACSDIVA v8.0 was used to collect data. FlowJo v10.4.2 was used to analyze data.
Cell population abundance	Populations were not sorted for downstream manipulation in this study.
Gating strategy	Cell lines were first gated on FSC-A vs SSC-A, then on FSC-A vs FSC-H for single cells, and then on negative staining for the live/dead ghost dye red 780 (Corning). Mean fluorescent intensity and histograms of H-2Kb (APC), H-2Db (FITC), and PD-L1 (PE-Cy7) were then calculated on this population.<br><br>Lung and lymph nodes were first gated on FSC-A vs SSC-A, then on FSC-A vs FSC-H for single cells, and then on negative staining for the live/dead ghost dye red 780. Intravascular cells were excluded by gating for IV CD45 negativity. In T cell depletion experiments, CD4 and CD8a positivity was then used to quantify numbers of these population. In M1-8 clonal mixing transplant experiments, neoantigen-specific T cells were gated by CD8a positivity, CD4 negativity, CD44 positivity, and tetramer/dextramer double positivity (PE and APC). TCF1 and GZMB staining was specifically assessed in this population.

☒ Tick this box to confirm that a figure exemplifying the gating strategy is provided in the Supplementary Information.
