# Supplementary material for: Mismatch repair deficiency is not sufficient to elicit tumor immunogenicity
Source: Nat Genet. 2023 Sep 14;55(10):1686–95. doi: 10.1038/s41588-023-01499-4 (PMC10562252; doi:10.1038/s41588-023-01499-4)

Source Data Fig. 7

Related to Extended Data Figure 1e

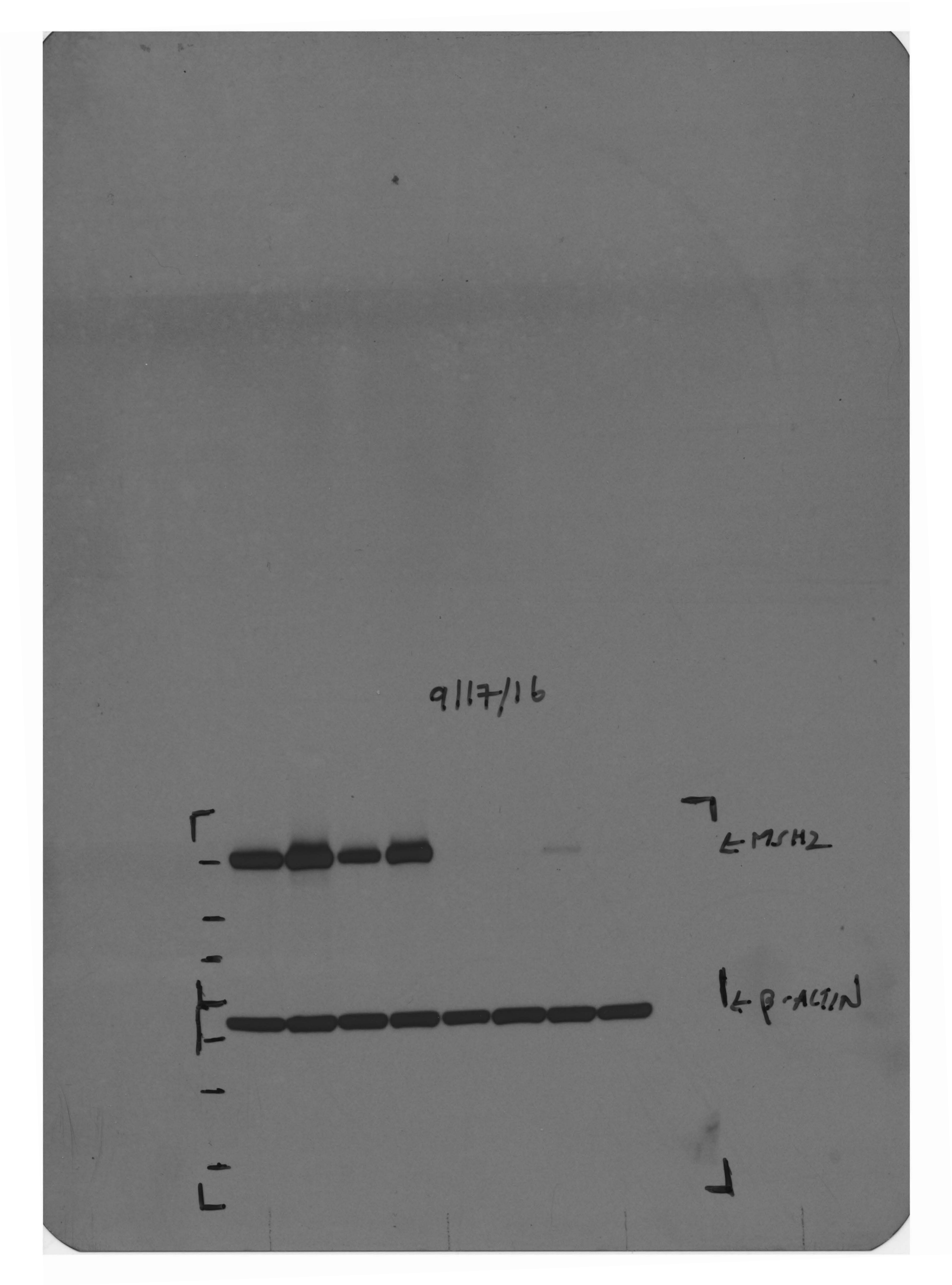

Source Data Fig. 7

Related to Extended Data Figure 1h

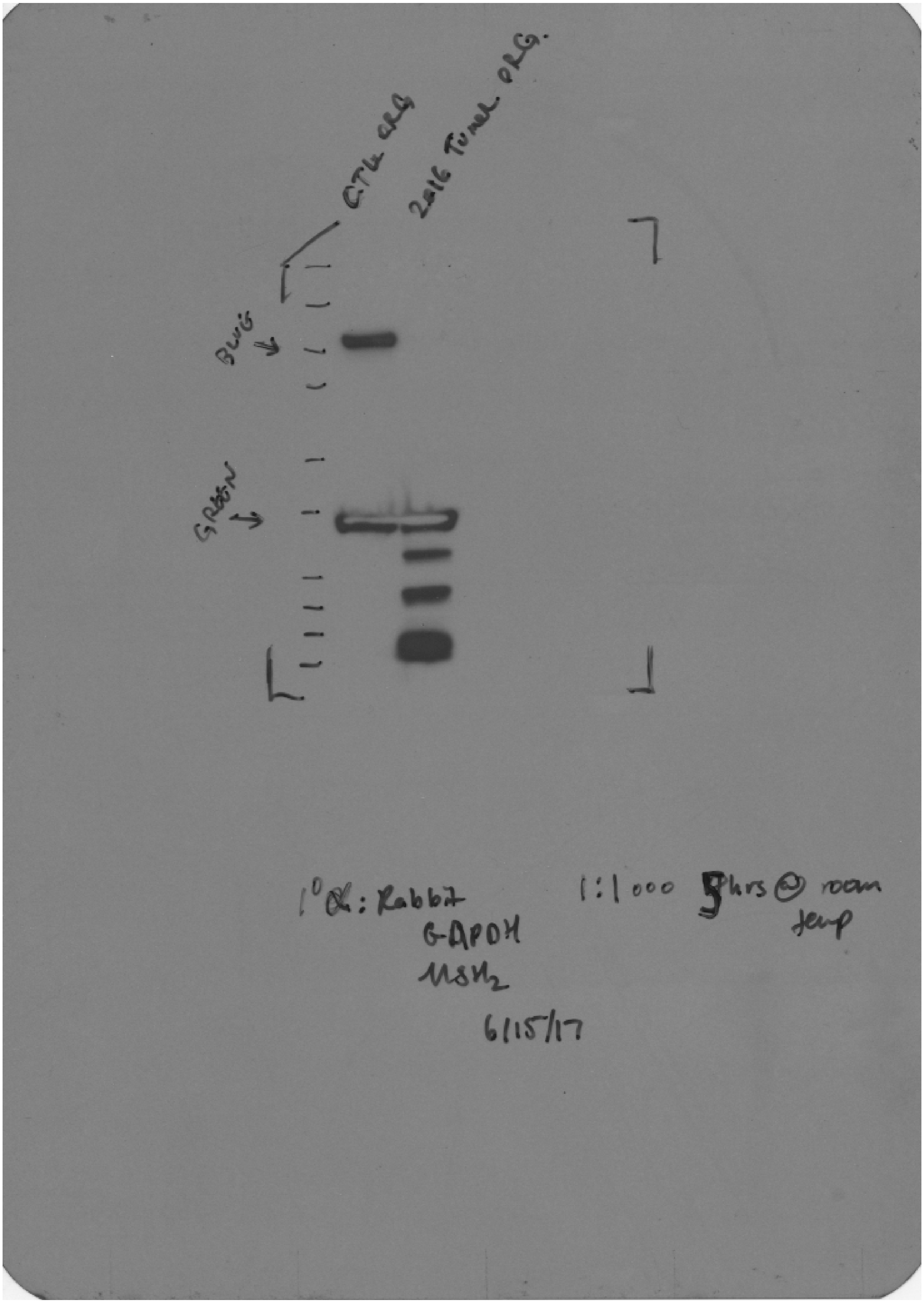

Source Data Fig. 7

Related to Extended Data Figure 3d

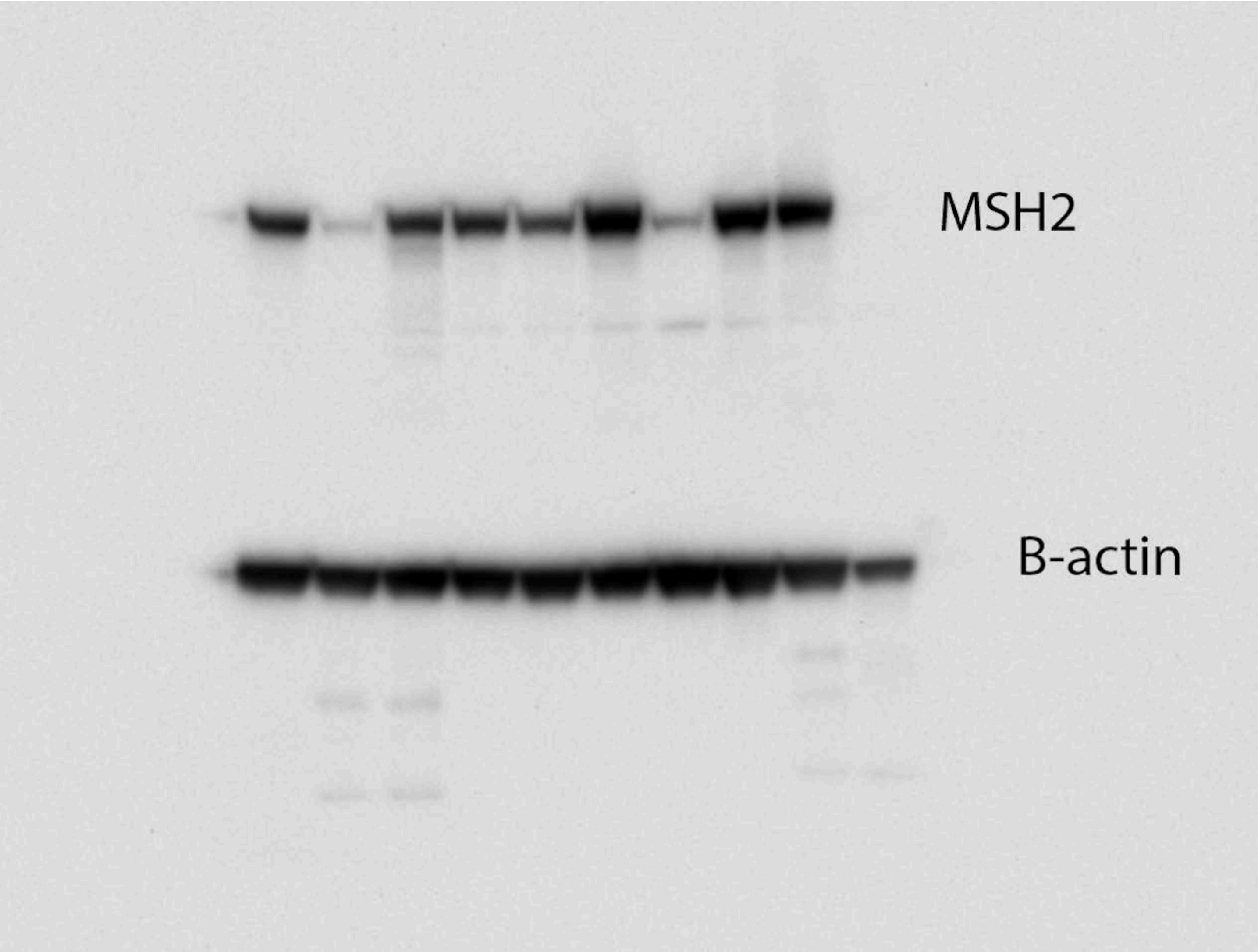

Supplement: Supplementary file 16 — Unprocessed western blots for Extended Data Figs. 1e,h,i and 3d. [file 41588_2023_1499_MOESM16_ESM.pdf]
